# Phosphorylation of Tyrosine 841 Plays a Significant Role in JAK3 Activation

**DOI:** 10.3390/life13040981

**Published:** 2023-04-10

**Authors:** Shengjie Sun, Georgialina Rodriguez, Yixin Xie, Wenhan Guo, Alan E. Lopez Hernandez, Jason E. Sanchez, Robert Arthur Kirken, Lin Li

**Affiliations:** 1 Computational Science Program, The University of Texas at El Paso, 500 W University Ave., El Paso, TX 79968, USA; 2 Department of Biological Sciences, The University of Texas at El Paso, 500 W University Ave., El Paso, TX 79968, USA; 3 Border Biomedical Research Center, The University of Texas at El Paso, 500 W University Ave., El Paso, TX 79968, USA; 4 Department of Information Technology, College of Computing and Software Engineering, Kennesaw State University, 1100 South Marietta Pkwy SE, Marietta, GA 30060, USA; 5 Department of Physics, The University of Texas at El Paso, 500 W University Ave., El Paso, TX 79968, USA

**Keywords:** JAK3, phosphorylation, tyrosine, Delphi, electrostatic potential, MMPBSA

## Abstract

Janus Kinase 3 (JAK3) plays a key role in the development, proliferation, and differentiation of various immune cells. It regulates gene expression by phosphorylation of Signal Transducers and Activators of Transcriptions (STATs) via the JAK/STAT pathway. Recently, we found a new JAK3 phosphorylation site, tyrosine 841 (Y841). The results showed that pY841 helps the kinase domain flip around the pseudo kinase domain, which may cause JAK3 conformational changes. It also reduces the size of the cleft between the N-lobe and the C-lobe of the JAK3 kinase domain. However, pY841 was found to enlarge the cleft when ATP/ADP was bound to the kinase. The increase in the cleft size suggested that pY841 enhanced the elasticity of the kinase domain. For unphosphorylated JAK3 (JAK3-Y841), the binding forces between the kinase domain and ATP or ADP were similar. After phosphorylation of Y841, JAK3-pY841 exhibited more salt bridges and hydrogen bonds between ATP and the kinase than between ADP and the kinase. Consequently, the electrostatic binding force between ATP and the kinase was higher than that between ADP and the kinase. The result was that compared to ADP, ATP was more attractive to JAK3 when Y841 was phosphorylated. Therefore, JAK3-pY841 tended to bind ATP rather than ADP. This work provides new insights into the role of phosphorylation in kinase activation and ATP hydrolysis and sheds light on the importance of understanding the molecular mechanisms that regulate the kinase function.

## 1. Introduction

The Janus Kinase (JAK) family comprises intracellular tyrosine kinases, including JAK1, JAK2, JAK3, and Tyrosine Kinase 2 (TYK2) [1,2]. They are involved in cytokine signaling via the phosphorylation of Signal Transducers and Activators of Transcription (STATs) [3] through the JAK/STAT pathway. When cytokines bind to their specific receptors, they cause the receptors to dimerize and activate JAK kinases to phosphorylate the receptor. This phosphorylation creates a docking site for STATs. Once the STATs are bound, they are also phosphorylated, dimerize, and translocate into the nucleus where they bind to DNA and regulate the expression of cytokine-responsive genes [4,5]. The JAK/STAT signaling pathway is critical for the normal functioning of hematopoietic cells, which are responsible for the production and maturation of blood cells. The dysregulation of this pathway has been associated with various hematological malignancies, including leukemia, lymphoma, and myeloproliferative neoplasms. Additionally, mutations in JAK genes have been identified in several types of cancers, such as myelofibrosis and acute lymphoblastic leukemia [6,7,8]. Understanding the mechanisms underlying the JAK/STAT pathway can provide insights into the development of new therapies for these diseases [9].

JAKs contain four fundamental domains: a carboxyl-terminal kinase domain (JH1), an anterior pseudokinase domain (JH2), a Src Homology 2 domain (SH2), and a four-point-one, Ezrin, Radixin, Moesin (FERM) domain. JH1 is the domain with catalytic ability, while JH2 lacks catalytic activity but possesses a regulatory function [2]. JAK1, JAK2, and TYK2 are ubiquitously found in different cell lineages [10,11], while JAK3 is expressed primarily in cells of the immune system [2,12,13,14]. In the JH1 domain, several phosphorylation sites were discovered that regulate the catalytic activity positively or negatively. Tyrosines 980 and 981 (Y980 and Y981) in JAK3 were discovered to be autophosphorylated. The kinase activity is positively regulated by phosphorylated tyrosine 980 (pY980) and negatively regulated by pY981 [15]. The phosphorylation of Y904 and Y939 was also shown to positively promote JAK3 signal transduction. The effects of the phosphorylation of the latter residues were found to be associated with the optimal usage of ATP and to promote STAT5 activity [16]. Correspondingly, autophosphorylation of homologous JAK2 sites was also found on Y868, Y972, and Y966. It proved the universality and importance of tyrosine phosphorylation in JAKs. Recently, we found and reported a new tyrosine phosphorylation site, JAK3-Y841, which positively regulates the catalytic activity in the activation of JAK3.

The biological evidence of the regulation of JAK by phosphorylation and dephosphorylation is solid and clear [13,16]. However, due to technical limitations, previous studies faced difficulties in providing structural details of the effects of these two processes, including the chemical and physical reasons for the activation of kinases. The challenges of revealing the biological process of the activation of a kinase are mainly due to the lack of quantitative analyses. Now, based on computational methods and advanced software, we can perform molecular dynamic (MD) simulations [17] to examine the impact of phosphorylation on protein structure. The phosphorylation effects on conformational changes, electrostatic forces, and binding energies can be further studied. In this work, we focused on the adenosine triphosphate/adenosine diphosphate (ATP/ADP) hydrolysis process and analyzed the binding between ATP/ADP and JAK3-JH1 in Y841/pY841 states. The results showed that the binding affinities between JH1-Y841 and ATP/ADP are similar, which may cause difficulties in releasing ADP. They also suggest that JH1-Y841 is not the optimal kinase state for ATP hydrolysis. By contrast, JAK3-pY841 has a higher binding affinity for ATP than for ADP, which is essential for the kinase to function normally by binding ATP and releasing ADP. Moreover, based on the full-length JAKs structure [18] and the autoinhibited state of JH1 and JH2 [19], we successfully modeled the full-length structure of JAK3 in the autoinhibited state. We found that pY841 also weakened the binding between JH1 and JH2, showing a higher sliding force on JH1. This could promote the JH1 flip to the activated position and expose the ATP binding pocket. 

## 2. Method

### 2.1. JAK3 Modeling

The autoinhibited/activated full-length JAK3 structure was built through SWISS-MOLDEL by the dimerized full-length JAK1 structure (PDB: 7T6F [18]) and autoinhibited JH1–JH2 dimer (PDB: 4OLI [19]). The phosphorylation of Y841 (pY841) was built by CHARMM-GUI [20], and the other residues were not phosphorylated. Autoinhibited full-length JAK3 structure with Y841 (FY841) and pY841 (FpY841) simulations were performed to study the effects of pY841 on JH1 in the autoinhibited state. JH1-Y841 and JH1-pY841 were separated from the whole structure to study the effects of pY841 on ATP/ADP hydrolysis. The JH1 domain with Y841/pY841 was combined with ATP/ADP to build 6 models, including two non-bound models (JH1-Y841 and JH1-pY841), two models bound to ATP (ATP-JH1-Y841 and ATP-JH1-pY841), and two models bound to ADP (ADP-JH1-Y841 and ADP-JH1-pY841). ATP or ADP was aligned into the ATP-binding pocket of the JH1 domain (the phosphate groups were outside, while adenosine was inside [21]). The model used for the alignment of ATP/ADP consisted of JAK3 JH1 with an inhibitor (PDB: 5TTV) [22]. We aligned the adenines of ATP/ADP to the inhibitor in 5TTV to form the JH1–ATP/ADP complexes.

### 2.2. Molecular Dynamics Simulation

For the simulations of each model, the force field applied was CHARMM36m [23]. The models were solvated by TIP3P [24] via CHARMM-GUI [20]. We used 150 mM KCl to ionize the system. Periodic boundary conditions and the long-range electrostatic interactions with particle mesh Ewald [25] were performed during the simulations. The temperature was set at 310 K, using a Langevin thermostat with a damping coefficient of 1/ps. The pressure was set to 1 atm using a Nosé–Hoover Langevin piston barostat with a decay period of 25 fs. The simulations were performed after a 10,000-step minimization. It included two steps: NPT equilibrium and NVT production. In equilibrium, the backbones of proteins and ATP/ADP were restrained. In production, all atoms were free except for the two residues in the N- and C-termini. The simulations were performed by NAMD 2.12 [17]. In the FY841 and FpY841 simulations, the 0.5 ns NPT equilibrium and 45 ns NVT production were performed. In the JH1-ATP/ADP simulations, the 0.5 ns NPT equilibrium and 100 ns NVT production were performed. 

### 2.3. Salt Bridges and Hydrogen Bonds

Salt bridge and hydrogen bond formation was analyzed by Visual Molecular Dynamics (VMD) [26]. The cutoff distance of the salt bridges was set as 4 Å, while the cutoff distance and the angle for the hydrogen bonds were set as 3.5 Å and 20° [27,28], respectively.

### 2.4. Topological Analysis

**Figure 1:**
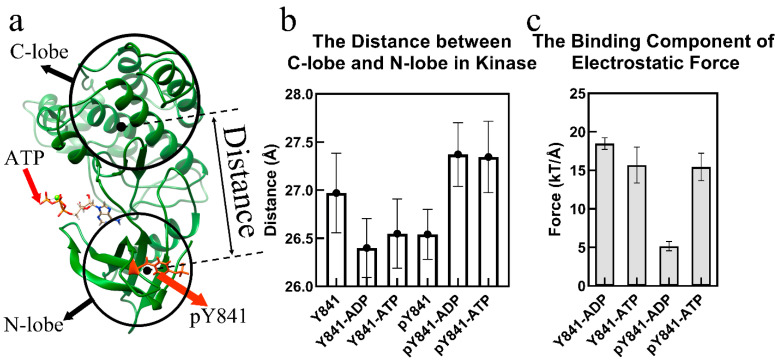
Phosphorylation of JH1-Y841 promotes an activated kinase domain state. (**a**) The ribbon diagram of the JAK3 JH1-pY841 domain with ATP. The JH1 cleft is the space between the N-lobe and the C-lobe. (**b**) Distance between the JH1 C-lobe and N-lobe in the simulations of JH1-Y841, JH1-pY841, ATP–JH1-Y841, ATP–JH1-pY841, ADP–JH1-Y841, ADP–JH1-pY841. (**c**) Binding component of the electrostatic force between the JH1 domain (Y841/pY841) and ADP/ATP (the error bar is based on the confidence interval of 0.95).

In the trajectories of the JH1–ATP/ADP simulations, we observed slight conformational changes in the N-lobe of the kinase domain. We calculated the distance between the mass centers of the N-lobe (T815-P906) and C-lobe (S907-S1100) (Figure 1a) by Structure-Man [29]. The distance represents the cleft size between the N-lobe and the C-lobe of the JH1 domain. The average and standard deviation of the distance in 300 frames (70 ns–100 ns) of 6 models (JH1-Y841, JH1-pY841, ATP–JH1-Y841, ATP–JH1-pY841, ADP–JH1-Y841, and ADP–JH1-pY841) were calculated to show the topological effects of the phosphorylation of Y841.

### 2.5. Electrostatic Analysis

The electrostatic potential of the N-lobe of JH1-Y841/pY841 with and without ATP/ADP at 0 ns and 100 ns were calculated by Delphi [30]. The negatively charged (red), positively charged (blue), and neutrally charged (white) surfaces of the N-lobe were depicted by Chimera [31]. The electrostatic forces between JH-1 and ATP/ADP were also calculated from 70 ns to 100 ns by DelphiForce [32]. The electrostatic forces between JH1 and JH2 in the FY841 and FpY841 models were calculated from 35 ns to 45 ns. The binding/sliding force was calculated using Equations (1) or (2):

(1) $$F_{binding}=F_{T}\cdot cos\alpha$$

(2) $$F_{sliding}=F_{T}\cdot sin\alpha$$

where *F_T_* represents the total electrostatic force between two subdomains/ligands, and α is the angle between the electrostatic force direction and the mass center connection of two subdomains/ligands (JH1–ATP/ADP or JH1–JH2). The charge and radius were assigned by pdb2pqr [33] from CHARMM36m [23]. The dielectric constant for protein and water were set as 2 and 80, respectively. Salt concentration, probe radius, filling ratio of the protein, and resolution were set as 150 mM, 1.4 Å, 0.70, and 2 grid/Å, respectively. The separations of JH1 from JAK3 were visualized by VMD [26]. 

### 2.6. MM/PBSA Free Binding Energy

The free energy was calculated by the last 10 ns frames of FY841 and FpY841. We separated JH1 from full-length JAK3 to calculate the binding free energy for JH1 and the FERM–SH2–JH2 complex. The free energy *E* was calculated by the sum of the Coulombic, van der Waals, polar, and nonpolar solvation binding energies (Equation (3)).

(3) $$E=E_{c}+E_{v}+E_{p}+E_{np}$$

where *E_c_* and *E_p_* represent the Coulombic binding energy and polar solvation energy, calculated by Delphi [30]. *E_v_* represents the van der Waals binding energy, calculated by NAMD 2.12 [17]. *E_np_* represents the nonpolar solvation energy, calculated by the solvent-accessible surface area (SASA) from Equation (4):

(4) $$E_{np}=\alpha \cdot SASA+\beta$$

where *E_np_* is the nonpolar solvation energy, *α* = 0.0054, and *β* = 0.92 kcal/mol. SASA was calculated using NACCESS [34].

The binding energy (Δ*E*) was calculated by the following equation (Equation (5)) [28,35,36]:

(5) $$\Delta E_{bind}=E_{complex}-E_{JH1}-E_{FERM+SH2+JH2}$$

where *E_complex_*, *E_JH_*_1_, and *E_FERM+SH+JH_*_2_ represent the free energy for the full-length JAK3, the JH1 domain, and the FERM–SH2–JH2 complex.

## 3. Results

### 3.1. JAK3-JH1–ATP/ADP Binding Process

A 100 ns simulation was performed for each of the JH1 domain states, including JH1-Y841, JH1-pY841, ATP–JH1-Y841, ATP–JH1-pY841, ADP–JH1-Y841, and ADP–JH1-pY841, and the stability of the conformations (with root-mean-square deviation <4 Å) was observed after 10 ns (Figure S1). Based on the last 30 ns simulations, the cleft size and electrostatic forces between the JH1 N-lobe and C-lobe were calculated. As shown in Figure 1b, the phosphorylation of Y841 resulted in a reduction of the JH1 cleft by around 0.5 Å, while the JAK3 JH1-pY841 cleft enlarged by 1 Å after binding with either ATP or ADP. Conversely, the ATP/ADP binding decreased the cleft size of JAK3-Y841 by about 0.6 Å. Despite the decrease in the JH1 cleft caused by pY841, ATP/ADP binding could still enlarge the cleft size, which allowed the substrate to enter and facilitated the transfer of gamma phosphate. The phosphorylation of Y841 reversed the conformational change caused by ATP/ADP binding. The wide range of cleft sizes indicated that the phosphorylation of Y841 increased the overall elasticity of the kinase domain. Additionally, the increased cleft size expanded the volume of the nucleotide-binding pocket and enhanced the binding force of ATP/ADP.

Figure 1c reveals that the binding forces between JH1 and ATP were similar in the presence or absence of Y841 phosphorylation (~15 kT/Å). However, the binding forces between JH1 without and with Y841 phosphorylation and ADP were significantly different. The binding force between JH1 without Y841 phosphorylation and ADP was ~18 kT/Å, which was slightly higher than that measured for the binding to ATP. This suggested that the kinase had difficulty releasing ADP. In contrast, after Y841 phosphorylation, the binding force between JH1 and ADP became much weaker, i.e., only ~5 kT/Å, significantly lower than that of the binding with ATP (ATP-JH1-pY841). The reduced binding force implies that release of ADP became easier, allowing a new ATP to bind and reactivate the catalytic activity. 

**Figure 2:**
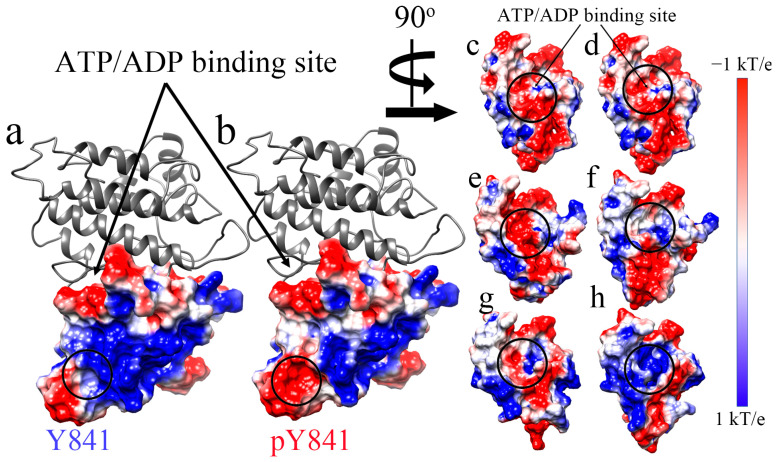
The electrostatic surface of the N-lobe of JAK3 JH before and after the simulations. (**a**,**c**) JH1-Y841 before the simulations. (**b**,**d**) JH1-pY841 before the simulations. (**e**) ATP–JH1-Y841 after the 100 ns simulation. (**f**) ATP–JH1-pY841 after the 100 ns simulation. (**g**) ADP–JH1-Y841 after the 100 ns simulation. (**h**). ADP–JH1-pY841 after the 100 ns simulation. The surface of the electrostatic potential is colored from −1 kT/e (red) to 1 kT/e (blue).

Subsequently, we also analyzed the electrostatic surface of the JH1 domain to visualize the effects of the phosphorylation of Y841 (Figure 2). We found that the pY841 state altered the positively charged surface area surrounding the site, as shown in Figure 2a,b. In the N-lobe of the JH1 ATP/ADP binding pocket, phosphorylation did not directly cause changes to the electrostatic surface, as shown in Figure 2c,d. Nevertheless, after the 100 ns simulations with ATP/ADP, the nucleotide-binding pocket showed significant differences. After the simulations with ATP, the binding pocket in JH1-Y841 was positively charged (Figure 2e circle), whereas the JH1-pY841 state was observed to be nearly neutral (Figure 2f circle). By contrast, the binding pocket was more neutral (Figure 2g circle) after the ADP–JH1-Y841 simulations, but more positive (Figure 2h circle) after the ADP–JH1-pY841 simulations. These electrostatic surface changes were caused by the conformational changes of peptide backbones and amino acid side chains. These changes could also contribute to the shifting size of the nucleotide-binding pocket. We observed that the phosphorylation of Y841 initially only affected a small area on the N-lobe, while the simulations amplified the effects on the binding pocket. The conformational changes induced by phosphorylation may create new surface charges or alter the distribution of existing charges, affecting the binding pocket’s overall charge distribution.

**Figure 3:**
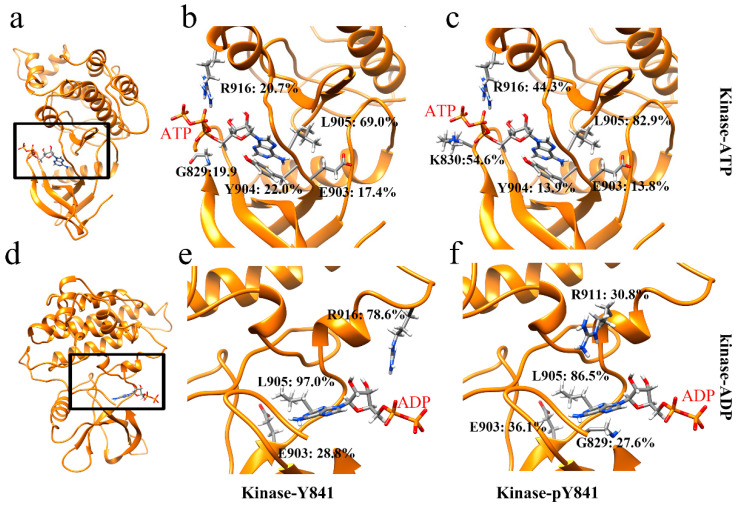
Ribbon diagram of high-occupancy (>10%) hydrogen bonds in (**a**) JH1 bound to ATP, (**d**) JH1 bound to ADP; hydrogen bonds between residues and nucleotides between (**b**) JH1-Y841 and ATP, (**c**) JH1-pY841 and ATP, (**e**) JH1-Y841 and ADP, (**f**) JH1-pY841 and ADP.

In order to gain a more comprehensive understanding of the effects of Y841 phosphorylation, we analyzed the salt bridges and hydrogen bonds at the intermolecular interfaces. Our analysis revealed that there was only one salt bridge, ATP–LYS830 (Figure S4), between ATP and JH1 in both states (Y841 and pY841). However, the occupancy of the salt bridge increased from 5.8% to 16.6% in the pY841 state. Correspondingly, the occupancies of the hydrogen bonds between ATP and JH1-Y841 (R916–ATP: 20.7%, L905–ATP: 69.0%) were much lower than those between ATP and JH1-pY841 (R916–ATP: 44.3%, L905–ATP: 82.9%) (Figure 3b,c). Specifically, the occupancies of R916–ATP and L905–ATP were 44.3% and 82.9%, respectively, in ATP–JH1-pY841, whereas they were only 20.7% and 69.0% in ATP–JH1-Y841 (Figure 3b,c). Notably, a new hydrogen bond between K830–ATP (occupancy of 54.6%) formed at the edge of the cleft in ATP–JH1-pY841, stabilizing ATP inside the nucleotide-binding pocket. In the ADP–JH1 Y841/pY841 simulations, no salt bridge formed between the kinase domain and ADP. However, the occupancy of the hydrogen bonds between ADP and JH1 was generally higher in the pY841 state compared to the Y841 state. Specifically, in the ADP–JH1-pY841 simulations, more residues could form hydrogen bonds with ADP, although most of them had very low occurrences. Only L905–ADP had an occupancy of 86.2%. On the other hand, in the ADP–JH1-Y841 simulations, L905–ADP and R916–ADP had occupancies of 97.0% and 78.6%, respectively. Notably, the hydrogen bond of R916–ADP in the ADP–JH1-Y841 state was located near the edge of the cleft, which might prevent ADP from being released and inhibit the recovery of the catalytic activity. Taken together, our data suggest that the binding force between ADP and JH1-Y841 was higher than that between ADP and JH1-pY841, further supporting the hypothesis that ADP was easily released after ATP hydrolysis from the Y841 phosphorylated kinase. These high-occupancy hydrogen bonds may serve as potential targets for therapeutic interventions.

### 3.2. pY841 Effects on Autoinhibited Full-Length JAK3

**Figure 4:**
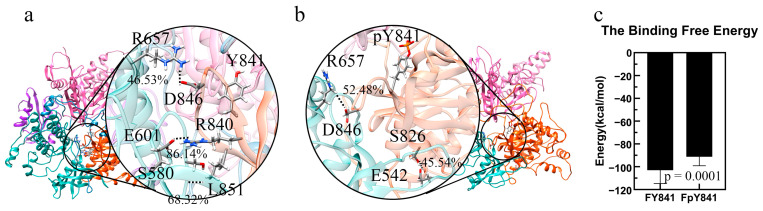
Ribbon diagram of high-occupancy (>30%) hydrogen bonds between JH1 and JH2 and binding free energy. FERM, SH2, JH2, and JH1 are colored pink, purple, blue, and orange, respectively. (**a**) High-occupancy hydrogen bonds in FY841. (**b**) High-occupancy hydrogen bonds in FpY841. (**c**) Binding free energy between JH1 and other domains (FERM, SH2, JH2) in full-length JAK3 with Y841(FY841) and pY841(FpY841).

The stability of the models of full-length, autoinhibited JAK3s with Y841 and pY841 (FY841 and FpY841) was assessed after 35 ns MD simulations, and both models were found to be stable (Figure S5). To gain further insights, we performed 10 ns MD simulations to analyze the force and energy involved. Our analysis showed that the high-occupancy hydrogen bonds of E601–R840 (86.14%) and S580–L851 (68.32%) disappeared after the phosphorylation of Y841, whereas the occupancy of the hydrogen bonds of R657–D846 increased from 46.53% to 52.48%. Moreover, a new hydrogen bond between S826 and E542 was formed, with 45.54% occupancy. The overall total occupancy of the hydrogen bonds decreased, indicating that the interactions between JH1-pY841 and JH2 were weaker than those between JH1-Y841 and JH2. Notably, key residues such as R841, L851, D846, and S826 are geometrically close to Y841, and the presence of pY841 strongly affects the adjacent residues and hydrogen bond formation. We also examined the binding free energy between JH1-Y841/pY841 and the FERM–SH2–JH2 complex and found that FpY841 had a much higher binding free energy (~10 kcal/mol) than FY841 in Figure 4c, suggesting that the presence of pY841 promoted the detachment of JH1 from the FERM–SH2–JH2 complex.

**Figure 5:**
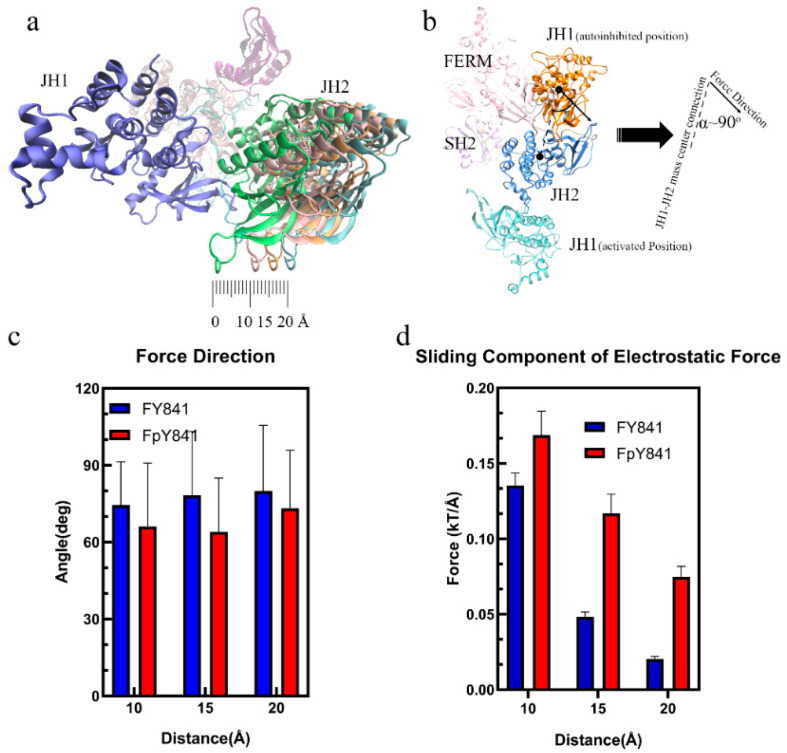
Interactions from FY841/FpY841 to JH1. (**a**) Diagram of the separation between JH1 and JH2. (**b**) Diagram of the angle (α) between the electrostatic force and the JH1–JH2 mass center connection. (**c**) Angle (α) between the JH1–JH2 mass center connection and the electrostatic force direction. (**d**) Sliding component of the electrostatic force between JH1 and JH2 at 10, 15, 20 Å.

The loss of hydrogen bonds and increased binding free energy caused by pY841 phosphorylation could lead to the detachment of JH1 from the autoinhibited JAK3, which is the first step towards activation. To further investigate the potential effects of Y841 phosphorylation on the conformational changes of JAK3, the JH1 and JH2 domains were separated by 10, 15, and 20 Å to simulate the initial steps of activation. The electrostatic forces between JH1 and JH2 were then analyzed to explore the potential continuous effects on JAK3 conformational changes. As shown in Figure 5a, the sliding force was the main component for sliding JH1 around JH2. Interestingly, the electrostatic force between JH1 and JH2 was nearly perpendicular to the JH1–JH2 mass center connection, as seen in Figure 5b,c. This perpendicular alignment indicated that the sliding force played a critical role in the sliding of JH1 around JH2, which is a crucial step in the activation process. It is also noteworthy that the JH1 in the activated JAK3 was on the other side of JH2 (Figure 5b), and the increased sliding force on JH1, caused by pY841, could contribute to the flipping of JH1 until the activated state was achieved. Overall, the simulation results suggest that Y841 phosphorylation may affect the detachment of JH1 from the autoinhibited JAK3 by reducing the hydrogen bonds and increasing the binding free energy. The increased sliding force on JH1 caused by pY841 may further contribute to the flipping of JH1 and the activation of JAK3. The electrostatic force between JH1 and JH2 was found to be perpendicular to the JH1–JH2 mass center connection, suggesting that the sliding force was the primary driving force in the JH1 sliding around JH2. These results provide new insights into the conformational changes of JAK3 and their regulation by Y841 phosphorylation, which may have important implications for the development of therapeutic interventions targeting JAK3.

## 4. Discussion

In this computational study, we conducted a topological analysis of the JAK3 JH1 kinase domain with and without ATP/ADP models to investigate the effects of pY841 on ATP hydrolysis. The results showed that the phosphorylation of Y841 initially decreased the cleft size of the JH1 domain, but dramatically increased the cleft size when ATP/ADP was bound to the kinase. This finding suggests that the phosphorylation of Y841 promotes the activation of JAK3 kinase by facilitating access to the ATP binding pocket. Furthermore, the analysis showed that the electrostatic binding forces between the kinase and ADP/ATP in the JH1-Y841 structure were similar. This similarity makes it difficult to bind ATP and release ADP, hindering the hydrolysis of ATP. However, after the phosphorylation of Y841, more salt bridges and hydrogen bonds formed between JH1 and ATP, which increased the electrostatic binding force on ATP. In contrast, ADP received a lower electrostatic binding force than ATP, making the release ADP easier. The increased binding force on ATP and the reduced binding force on ADP, induced by the phosphorylation of Y841, thus lowered the difficulty for releasing ADP and facilitated the recovery of the kinase catalytic activity. These findings support the idea that pY841 encourages an active JAK3 kinase state by promoting the optimal usage of ATP. To be noted, the phosphorylation of Y904 and Y939 was shown to positively promote the optimal usage of ATP [16], which suggests that these residues play a critical role in the activation of the JAK3 kinase. One of our key findings was that E903, Y904, and L905 contributed over 100%-occupancy hydrogen bonds and that the phosphorylation of Y841 strongly affected the hydrogen bonds between these key residues and ATP/ADP. This is consistent with previous experimental works [16] and highlights the critical importance of the phosphorylation of Y841 in JAK3 kinase activity. Initially, we observed that pY841 decreased the positively charged surface area surrounding the phosphorylated site without impacting the electrostatic surface of the nucleotide-binding pocket. However, our simulations revealed that over time, more areas in the binding pocket became negatively/positively charged in the pY841 state. This led to the formation of more salt bridges and hydrogen bonds between JH1 and ATP, while fewer salt bridges and hydrogen bonds were formed between JH1 and ADP. As a result, the electrostatic binding force between JH1 and ADP was reduced, making it easier for ADP to be released. This likely encouraged the kinase to recover catalytic activity. Furthermore, we also found that pY841 broke many hydrogen bonds between JH1 and JH2, which are key residues to stabilize JH1 in the autoinhibited states. The separated JH1-pY841 received a higher sliding force compared to JH1-Y841, suggesting that pY841 may contribute to the flip of JH1 into the activated position. This is illustrated in Figure 5b of our study.

Overall, our work provides new insights into the role of pY841 in JAK3 kinase activity and sheds light on the importance of understanding the molecular mechanisms that regulate the kinase function.

## 5. Conclusions

The phosphorylation of the tyrosine residue Y841 on the Janus kinase (JAK) protein initiates a cascade of events that ultimately leads to the activation of the protein kinase. Specifically, the phosphorylation of Y841 breaks the hydrogen bonds between the JH1 and JH2 domains, which reduces the binding affinity between them. As a result, the JH1 domain detaches from JH2 and flips around it, exposing the ATP-binding pocket. During this process, the sliding force acting on JH1-pY841 is increased, promoting its movement to the activated state. The analysis of the ATP/ADP-binding pockets in the JAK kinase revealed that the phosphorylation of Y841 only negatively charges a small portion of the kinase and does not directly affect the ATP/ADP-binding pockets. However, long-term simulations of 100 ns showed that phosphorylation indirectly affects the binding pockets. Steric effects and negative charges of phosphorylation cause changes in the negatively and positively charged areas of JH1–ATP/ADP that ultimately affect the cleft size and binding force. The highly charged area of JH1–ATP/ADP not only increases the cleft size when ATP/ADP is bound to the kinase but also strengthens the electrostatic binding force on ATP while weakening it on ADP. This reduced force on ADP facilitates the release of ADP, which further facilitates the recovery of the kinase catalytic activity. On the other hand, JH1-pY841–ATP showed a higher occupancy of hydrogen bonds, contributing to the attraction of ATP. Moreover, JH1-pY841–ADP showed fewer hydrogen bonds than JH1-pY841–ATP, facilitating the release of ADP after hydrolysis. The reduced hydrogen bonding in JH1-pY841–ADP leads to a lower binding force between ADP and the JAK kinase, which makes it easier for ADP to be released after hydrolysis. Overall, the phosphorylation of Y841 plays a critical role in the activation of the JAK kinase. It initiates the separation of the JH1 and JH2 domains, promoting the movement of JH1-pY841 to the activated state and exposing the ATP-binding pocket. Furthermore, phosphorylation indirectly affects the binding pockets by changing the negatively and positively charged areas of JH1–ATP/ADP, which ultimately affects the cleft size and binding force. These changes facilitate the release of ADP after hydrolysis, which further promotes the recovery of the kinase catalytic activity.

## Data Availability

The data can be shared up on request.

## Supplementary Materials

The following supporting information can be downloaded at: https://www.mdpi.com/article/10.3390/life13040981/s1, Figure S1: RMSD of the kinase Y841 and pY841 and that with ADP or ATP; Figure S2: Cleft size of the kinase Y841 and pY841, and that with ADP or ATP; Figure S3: Binding components of the electrostatic force between the kinase and ADP/ATP in Y841, pY841; Figure S4: Distances between the salt bridge pairs LYS830/ATP in Y841 and pY841. The occupancy of LYS830/ATP is 5.8%, while that in pY841 is 16.6%; Figure S5: RMSD of full-length JAK3 with non- phosphorylated and phosphorylated Y841 (FY841 and pY841) in the auto-inhibited state; Figure S6: RMSF of full-length JAK3 with non-phosphorylated and phosphorylated Y841 (FY841 and FpY841) in the auto-inhibited state.
