# Supplementary material for: Phosphorylation of Tyrosine 841 Plays a Significant Role in JAK3 Activation
Source: Life (Basel). 2023 Apr 10;13(4):981. doi: 10.3390/life13040981 (PMC10141632; doi:10.3390/life13040981)
Supplement: Supplementary file 1 [file life-13-00981-s001.zip › life-2257164-supplementary.pdf]

Phosphorylation of Tyrosine 841 Plays a Significant Role in JAK3 Activation

Shengjie Sun[1], Georgialina Rodriguez[2,3], Yixin Xie[1,4], Wenhan Guo[1], Alan E Lopez Hernandez[1], Jason E Sanchez[1], Robert Arthur Kirken[2,3], Lin Li[1,5,*]

Correspondence to: Lin Li (e-mail: lli5@utep.edu)

[1] Computational Science Program, University of Texas at El Paso, 500 W University Ave, TX, 79968, USA;

[2] Department of Biological Sciences, the University of Texas at El Paso, 500 W University Ave, TX, 79968, USA;

[3] Border Biomedical Research Center, the University of Texas at El Paso, 500 W University Ave, TX, 79968, USA;

[4] Department of Information Technology, College of Computing and Software Engineering, Kennesaw State University, 1100 South Marietta Pkwy SE, Marietta, GA 30060;

[5] Department of Physics, the University of Texas at El Paso, 500 W University Ave, TX, 79968, USA;

**To whom correspondence should be addressed.*

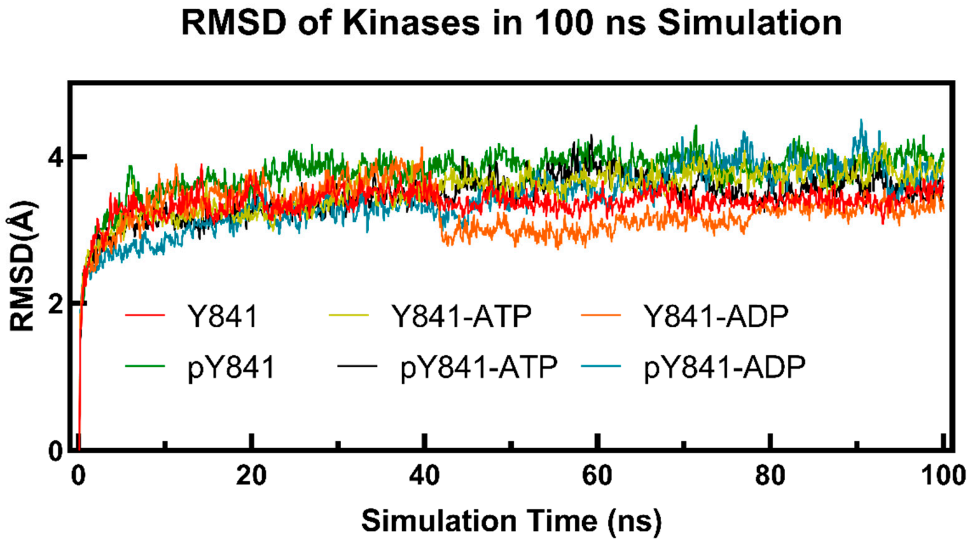

Figure S1: RMSD of the kinase Y841, pY841, and that with ADP or ATP.

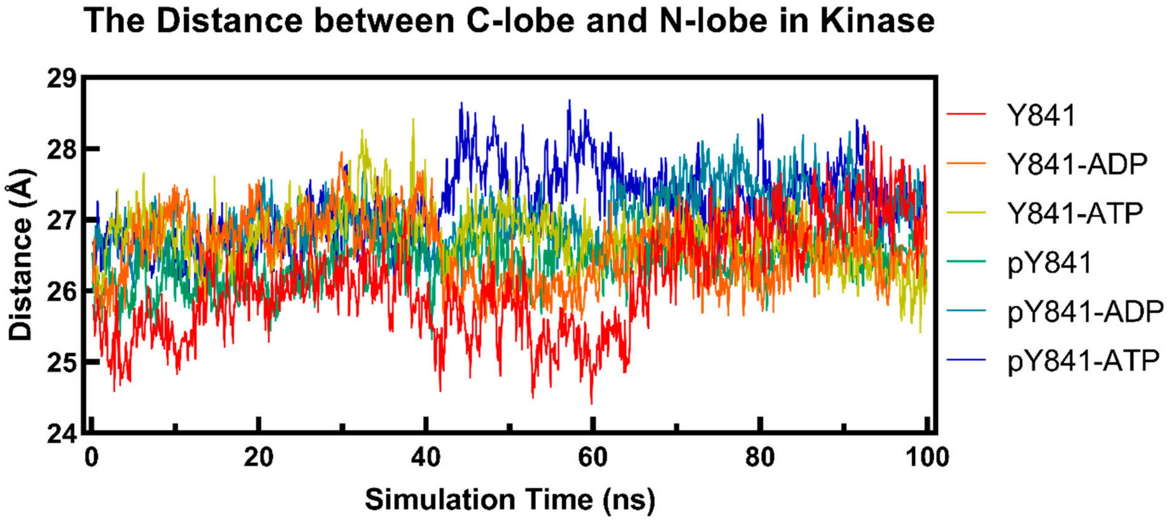

Figure S2: Cleft size of kinase Y841, pY841, and that with ADP or ATP.

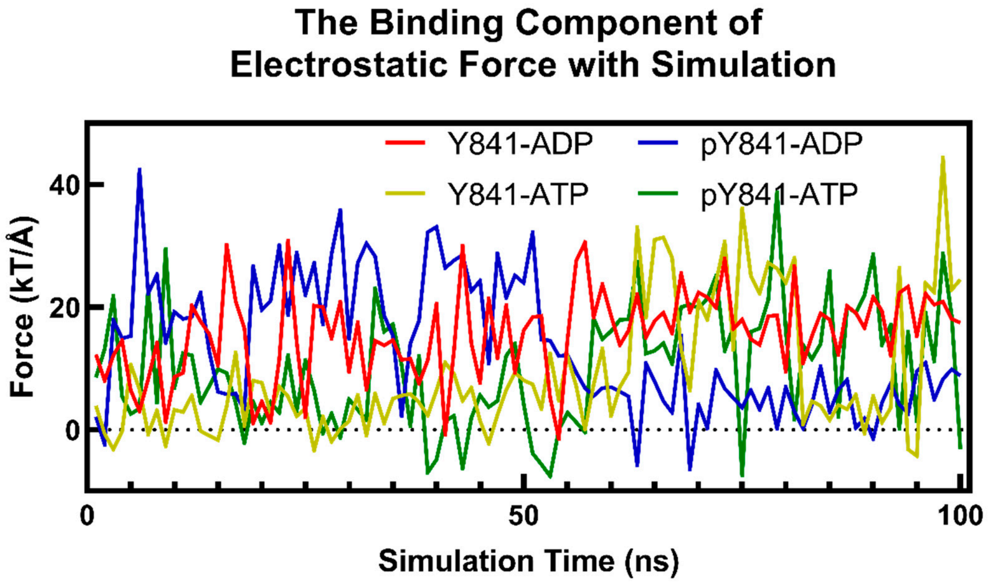

Figure S3: Binding components of electrostatic force between kinase and ADP/ATP in Y841, pY841.

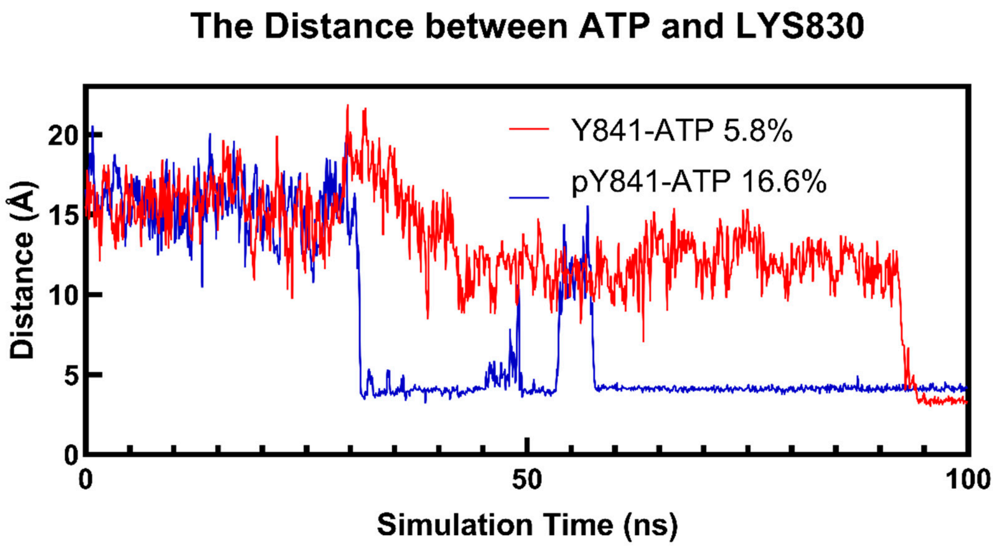

Figure S4: Distances between the salt bridge pairs LYS830/ATP in Y841 and pY841. The occupancy of LYS830/ATP in 5.8% while that in pY841 is 16.6%.

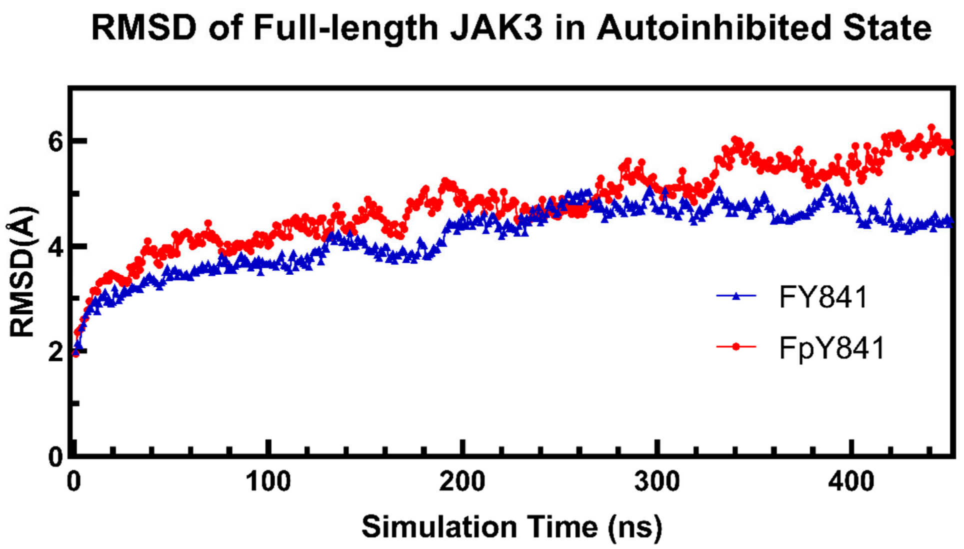

Figure S5: RMSD of full length JAK3 with non- phosphorated and phosphorated Y841 (FY841 and pY841) in auto-inhibited state

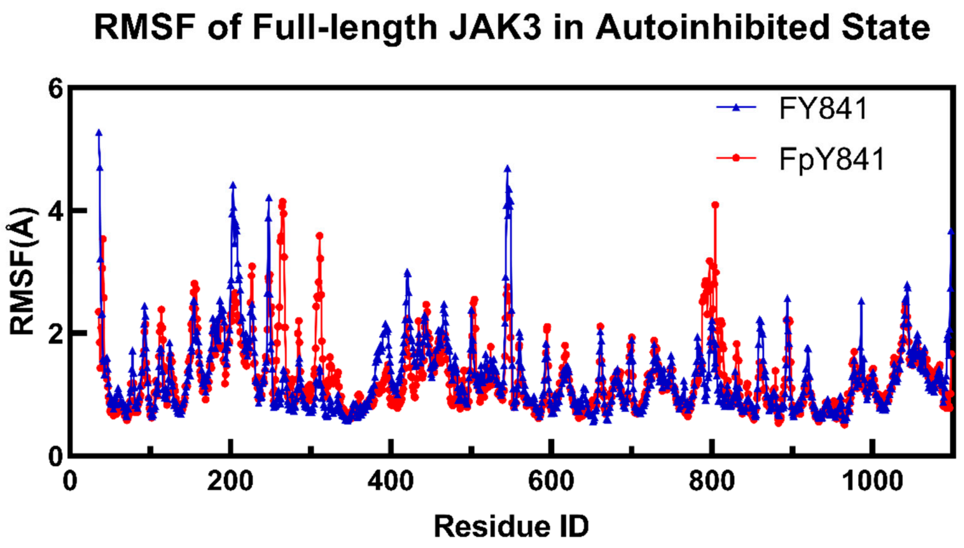

Figure S6: RMSF of full length JAK3 with non-phosphorated and phosphorated Y841 (FY841 and FpY841) in auto-inhibited state
